# Minimally invasive pediatric surgery: Our experience

**DOI:** 10.4103/0971-9261.43800

**Published:** 2008

**Authors:** K. Saravanan, V. Kumaran, G. Rajamani, S. Kannan, N. Venkatesa Mohan, M. Nataraj, R. Rangarajan

**Affiliations:** Department of Pediatric Surgery, Coimbatore Medical College Hospital, Coimbatore, India

**Keywords:** Laparoscopy, pediatric, thoracoscopy

## Abstract

**Aim:**

Departmental survey of the pediatric laparoscopic and thoracoscopic procedures.

**Materials and Methods:**

It is a retrospective study from January 1999 to December 2007. The various types of surgeries, number of patients, complications and conversions of laparoscopic and thoracoscopic procedures were analyzed.

**Results:**

The number of minimally invasive procedures that had been performed over the past 9 years is 734, out of which thoracoscopic procedures alone were 48. The majority of the surgeries were appendicectomy (31%), orchiopexy (19%) and diagnostic laparoscopy (16%). The other advanced procedures include laparoscopic-assisted anorectoplasty, surgery for Hirschprung’s disease, thoracosocpic decortication, congenital diaphragmatic hernia repair, nephrectomy, fundoplication, etc. Our complications are postoperative fever, bleeding, bile leak following choledochal cyst excision and pneumothorax following bronchogenic cyst excision. A case of empyema thorax following thoracoscopic decortication succumbed due to disseminated tuberculosis. Our conversion rate was around 5% in the years 1999 to 2001, which has come down to 3% over the past few years. Conversions were for sliding hiatus hernia, nephrectomy, perforated adherent appendicitis, Meckel’s diverticulum, thoracoscopic decortication and ileal perforation.

**Conclusion:**

The minimally invasive pediatric surgical technique is increasingly accepted world wide and the need for laparoscopic training has become essential in every teaching hospital. It has a lot of advantages, such as less pain, early return to school and scarlessness. Our conversion rate has come down from 5% to 3% with experience and now we do more advanced procedures with a lower complication rate.

## INTRODUCTION

After the success of minimally invasive surgical techniques in adults, application in pediatric patients was the next logical step. The use of these techniques in young children spread slowly, however, because the surgical instruments had to be miniaturised, the learning curve was relatively long and safe and reliable anesthetic procedures had to be developed to ensure good tolerance to pneumoperitoneum and pneumothorax. Today, it is essential that every pediatric surgeon must have adequate training in minimally invasive surgery as it has many advantages to the patient. Our experience is shared below.

## MATERIALS AND METHODS

It is a retrospective study of data from January 1999 to December 2007. The variables included in the study are the type of procedures, quantity of the procedures, various complications and conversion rates.

## RESULTS

**Figure 1:**
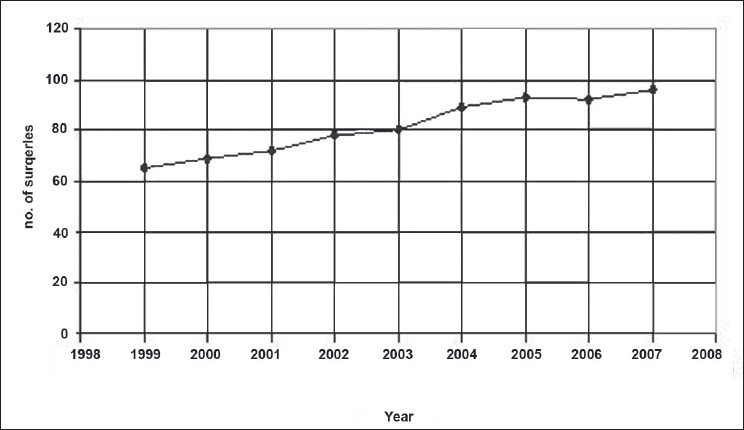
Year-wise statistics

**Figure 2:**
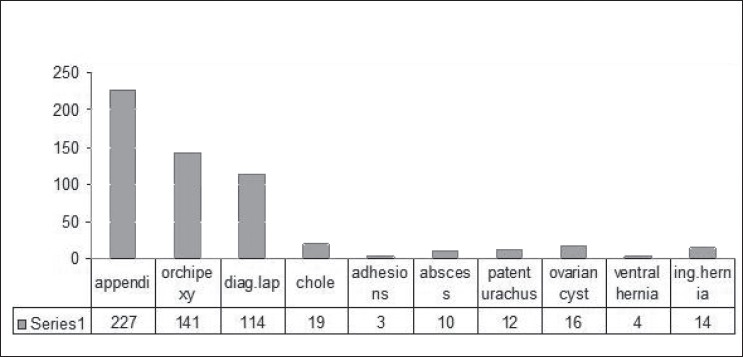
Basic laparoscopic surgery

**Figure 3:**
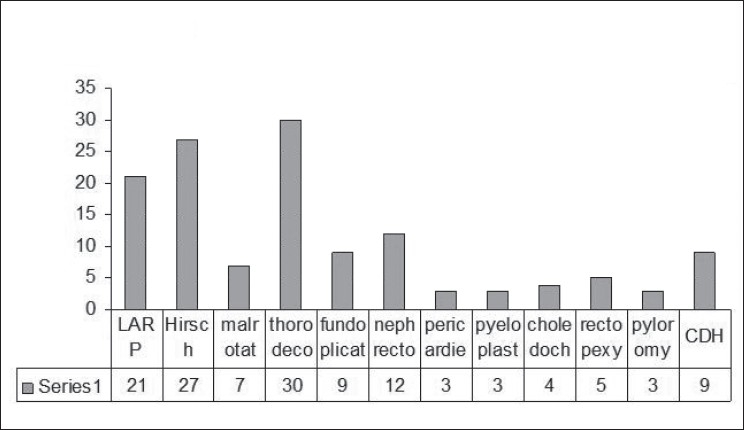
Advanced laparoscopic surgery

We have performed 734 cases from January 1999 to December 2007 [Figure 1], of which there were 65 cases in the year 1999 and later progressing to 96 cases in the year 2007. There was an average increase of 10% per year. Over the past 9 years, 48 thorocoscopic procedures were performed. The majority of the cases were appendicectomy [227 (31%)], orchiopexy [141 (19 %)] and diagnostic laparoscopy [114 (16%)] [Figure 2]. Advanced laparoscopic procedures include rectopexy (5 cases), malrotation (7), hiatus hernia (9), anorectal anomaly (21), nephrectomy (12), Hirschprung’s disease (27), adrenalectomy, pyeloplasty (3), choledochal cyst (4), infantile hypertrophic pyloric stenosis (3), hydatid cyst and colovaginoplasty (2). Thoracoscopic procedures started with biopsy and subsequently progressed to thoracoscopic decortication (30 cases), congenital diaphragmatic hernia repair (9), bronchogenic cyst excision, lobectomy (3) and thoracoscopic pericardiectomy (3) [Figure 3].

Our conversion rate was around 5% in the years 1999–2000, which has come down to 3% from the year 2005 onwards. Conversions were for sliding hiatus hernia, ileal perforation, perforated adherent appendicitis, Meckel’s diverticulum and laparoscopic nephrectomy. Two cases of thoracoscopy for empyema thoracis were converted to thoracotomy due to thick fibrous peel.

**Figure 4:**
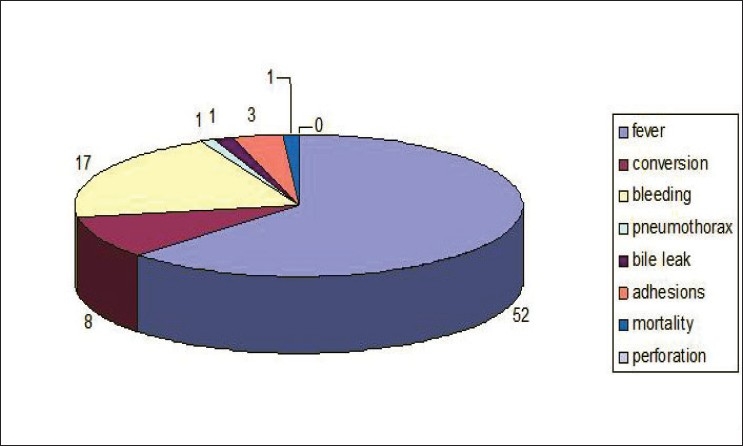
Complications

Our postoperative complications [Figure 4] were fever (15%) in a majority of the patients. One case of choledochal cyst developed bile leak, which was reopened, and Roux–En–Y hepaticojejunostomy was performed. A case of bronchogenic cyst that developed pneumothorax after discharge improved with ICD. One mortality occurred in a case of thoracoscopic decortication due to disseminated tuberculosis.

## DISCUSSION

Numerous case series on minimally invasive pediatric surgery have been published in the recent years.[1–4] This paper highlights our institutional experience over the past 9 years, which is a high volume postgraduate teaching institute. The spectrum of minimally invasive procedures is wide, ranging from basic to advanced procedures. In the German pediatric population,[5] laparoscopic procedures were offered for 96% of abdominal surgeries and in 74% of thoracic procedures, indicating that principally minimally invasive technique has been widely accepted. Data on the learning curve in minimally invasive pediatric surgery is lacking. The learning curve for lap cholecystectomy in adults ranges from 10 to 75 procedures.[6] Chang et al.[7] showed that the duration of the operation correlated negatively with the experience of the surgeon. Our youngest baby who underwent thoracoscopic diaphragmatic hernia repair was 5 days old. A camera port was placed through the 5^th^ intercostal space in the mid axillary line and other two ports based on the site of pathology, whether involving the diaphragm or the pleural cavity. For laparoscopy, we used the umbilical port for the camera and two working ports depending on the pathology. From our experience, the safety of minimally invasive surgery has increased with time. Laparoscopy is a great boon to the patients due to less pain, more comfort and early return to school, decreasing agony to parents and children. It is important to train the postgraduates and residents so that future laparoscopic surgery is widely available for the common population with a lesser complication and a higher safety margin.

## CONCLUSION

Laparoscopic surgery in children certainly carries many advantages. These include less pain, rapid recovery, shorter hospital stay, less wound complications and better cosmesis. The laparoscopic technique has also revolutionized the management of some disease conditions in children such as undescended testes, Hirschsprung’s disease, imperforate anus and gastroesophageal reflux. Although data from large prospective series on the safety and efficacy of laparoscopic techniques in young infants are still lacking currently, preliminary data provide convincing evidence that the technique is very promising. With increase in experience, rapid advancement in technology and better understanding of the physiological impact of the procedure, what is difficult to accomplish today may become a straightforward routine in the near future, and it is almost certain that laparoscopic surgery in infants and young children will become more widely accepted.
